# Induction of gastric cancer cell adhesion through transforming growth factor-beta1-mediated peritoneal fibrosis

**DOI:** 10.1186/1756-9966-29-139

**Published:** 2010-10-29

**Authors:** Zhi-Dong Lv, Di Na, Fu-Nan Liu, Zong-Min Du, Zhe Sun, Zhen Li, Xiao-Yang Ma, Zhen-Ning Wang, Hui-Mian Xu

**Affiliations:** 1 Department of Surgical Oncology, The First Hospital of China Medical University, Shenyang 110001, Liaoning Province, China; 2 Department of Surgical Oncology, General Hospital of Benxi Iron and Steel, Benxi 117000, Liaoning Province, China; 3 Department of General Surgery, The Fourth Hospital of China Medical University, Shenyang 110032, Liaoning Province, China

## Abstract

**Background:**

Peritoneal dissemination is one of the main causes of death in gastric cancer patients. Transforming growth factor-beta1 (TGF-β1), one of the most potent fibrotic stimuli for mesothelial cells, may play a key role in this processing. The purpose of this study is to elucidate the effects of TGF-β1 on regulation of gastric cancer adhesion to mesothelial cells.

**Methods:**

Peritoneal tissues and peritoneal wash fluid were obtained for hematoxylin and eosin staining or ELISA to measure fibrosis and TGF-β1 levels, respectively. The peritoneal mesothelial cell line, HMrSV5, was used to determine the role of TGF-β1 in regulation of gastric cancer cell adhesion to mesothelial cells and expression of collagen, fibronectin, and Smad 2/3 by using adhesion assay, western blot, and RT-PCR.

**Results:**

The data showed that TGF-β1 treatment was able to induce collagen III and fibronectin expression in the mesothelial cells, which was associated with an increased adhesion ability of gastric cancer cells, but knockdown of minimal sites of cell binding domain of extracellular matrix can partially inhibit these effects.

**Conclusion:**

Peritoneal fibrosis induced by TGF-β1 may provide a favorable environment for the dissemination of gastric cancer.

## Background

Gastric cancer is a significant health problem in most developing countries, including China, and is the second leading cause of cancer death worldwide [1]. The exact cause of gastric cancer has been elusive and the risk factors identified to date are variable and include *helicobacter pylori *infection, tobacco smoking, alcohol consumption and unhealthy diet. Gastric cancer usually remains undiagnosed until late stages due to asymptomatic or nonspecific presentation in its early stages; by the time clinical symptoms manifest, the cancer has generally metastasized to other parts of the body [1-4]. Peritoneal carcinomatosis frequently occurs at the later stages of gastric carcinoma, especially after surgery [2-4], which refers to the peritoneal metastatic cascade of gastric cancer and significantly contributes to gastric cancer-related mortality. To date, the mechanisms by which gastric carcinoma undergoes peritoneal carcinomatosis has not yet been specified.

Stephen Paget's 'seed and soil' theory of tumor metastasis may provide a clue useful for further investigation. This theory stated that the sites where metastasis occurs are defined not only by the tumor cells (seed) but also by the local microenvironment of the metastatic site (soil) [5]. In other words, the specific site of cancer cell metastasis is not simply due to anatomic location of the primary tumor or proximity to secondary sites but rather, it involves interactions between tumor cells and the local microenvironment at the secondary site [6]. Therefore, peritoneal carcinomatosis may occur as the peritoneal stroma environment promotes tumor cells to attach to the peritoneal mesothelium by providing various growth factors and chemokines that promote tumor metastasis [7]. This process is established by the interactions between extracellular matrix associated proteins and signals produced by mesothelial cells and the corresponding adhesion molecules from tumor cells [8].

Extracellular matrix(ECM) that contains collagen, laminin, fibronectin and hyaluronic acid provides ligands for b1-integrin and CD44 h and is known to participate in the peritoneal dissemination of cancer cells [9]. Transforming growth factor-β, a family of 25 kDa homodimeric multifunctional regulatory peptides, possesses a number of biological functions, including extracellular matrix production and maturation [10]. TGF-β1 is one of the most potent fibrosis stimuli of mesothelial cells [11]; increasing evidence has suggested that TGF-β1 can induce synthesis of extracellular matrix proteins and has been implicated as the key mediator of fibrogenesis in various tissues [12]. In our previous study, we demonstrated that the TGF-β1 level in peritoneal lavage fluid is correlated with peritoneal metastasis of gastric cancer. Other studies have shown that TGF-β1 is able to stimulate invasion and adhesion of scirrhous gastric cancer cells to the peritoneum, resulting in an increase in peritoneal dissemination of tumor cells [13-16]. However, little is known about the underlying mechanisms that regulate this activity. Adhesion polypeptides are located in the cell binding domain of ECM components, such as fibronectin, laminin, and collagen, and can bind to specific cell surface cellular adhesion molecules (CAM) known as integrins for cell-to-ECM adhesion. However, the common and characteristic RGD (Arg-Gly-Asp sequences) have been found to selectively block the binding of tumor cells to ECM, and to consequently inhibit metastasis [17].

In this study, we hypothesized that disseminated gastric cancer cells secrete abundant inflammatory factors, such as TGF-β1, in order to induce peritoneal fibrosis and production of extracellular matrix to generate a suitable microenvironment (peritoneal fibrosis) for their peritoneal dissemination. We first investigated histopathologic changes in the peritoneum and TGF-β1 concentrations in peritoneal lavage fluid. We then determined the effects of TGF-β1 on the function of human peritoneal mesothelial cells (HPMCs) and of microenvironment changes on the ability of gastric cancer cells to attach to mesothelial cells in the early stages of peritoneal dissemination.

## Materials and methods

### Reagent and Instrument

Total Smad-2/3, phosphorylated- Smad2 and phosphorylated- Smad3 antibodies, as well as second antibodies were purchased from Santa Cruz Biotechnology Inc, USA. Calcein-AM was brought from CALBIOCHEM, UK. RGD (Arg-Gly-Asp), which is the cell binding domain of the ECM, were obtained from Sigma (Osaka, Japan). Dulbecco's modified Eagle's medium and fetal calf serum(FCS) were purchased from GIBCOBRL, USA. Human TGF-β1 was obtained from Sigma, USA. human TGF-β1 ELISA kit (R&D, Minneapolis, MN, USA). Hematoxylin and eosin and Masson stain kit(Santa Cruz Biotechnology Inc, USA). Phasecontrast microscope (Japan Nikon). Spectrofluorometer (Japan Olympus, Japan) were employed. Other laboratory reagents were obtained from Sigma, USA.

### Cell line and culture

A human peritoneal mesothelial cell line HMrSV5 was kindly provided by Prof. Youming Peng of the Second Hospital, Zhongnan University, Changsha, PR China and Prof. Pierre RONCO, Hospital TENON, Paris, France. This cell line was established after infection of a fully characterized primary culture of human peritoneal mesothelial cells with an amphotropic recombinant retrovirus that encodes SV40 large-T Ag under control of Moloney virus long terminal repeat. An undifferentiated human gastric carcinoma cell line, HGC-27, was obtained from the Cancer Research Institute of Beijing, PR China, and HSC-39 cell line was derived from the ascites of a signet ring cell gastric carcinoma, which was obtained from the Department of Medicine, Kyushu University, Japan. These cell lines were cultivated in T75 tissue culture flasks in DMEM supplemented with 10% fetal calf serum, 100 U/ml penicillin, 100 μg/ml streptomycin, 2 mM L-glutamine, and 20 mM hydroxyethyl piperazine ethanesulfonic acid (HEPES). Cultures were grown at 37°C in a humidified 5% CO2 and 95% air incubator.

### Tissue samples

Human peritoneum tissue samples were obtained from 36 gastric cancer patients and 6 benign disease patients who underwent surgery in the First Affiliated Hospital of China Medical University between March 2009 and October 2009. These tissue specimens were taken from the lower anterior abdominal wall. No patients had received any form of radiation or chemotherapy before surgery. The local institutional review board approved our protocol for use of patient samples; all patients provided written informed consent prior to participation in the study. All histology slides were staged and classified according to the UICC new TNM staging (7th edition). The peritoneal tissues were directly obtained from the surgical suite and immediately fixed in 10% buffered formalin and then embedded in paraffin. Sections (5 μm) were prepared and stained with hematoxylin and eosin and Masson stain. The thickness of the submesothelial extracellular matrix was determined after the tissue sections were hematoxylin and eosin and Masson staining, the average of 10 independent measurements was calculated for each section and then the data were summarized.

### ELISA detection of TGF-β1 levels in the peritoneal lavage fluid

The peritoneal lavage fluid was also collected from each patient. Briefly, during laparotomy, 100 mL physiological saline was injected into the right upper quadrant or the Douglas pouch and approximately 60 mL were retrieved. The peritoneal lavage sample was immediately centrifuged at 2000 rpm for 10 min at room temperature, and stored at -80°C until use. The TGF-β1 levels were then assayed with a human TGF-β1 ELISA kit according to the manufacturer's instructions. The data on the TGF-β1 protein levels were summarized as mean ± SE of each sample.

### Semi-quantitative reverse transcription polymerase chain reaction (RT-PCR)

The cells were grown to subconfluence and then starved for 15 h in serum-free medium to attain quiescence. Afterwards, the cells were washed twice with PBS and cultured in either serum-free medium (control) or serum-free plus 2 or 10 ng/mL of TGF-β1 (experimental group) for up to 72 h. Total RNA was isolated from these cells using the TRIzol reagent according to the manufacturer's instructions. One microgram of the total cellular RNA was then reverse-transcribed into cDNA for PCR amplification using a kit from Sigma. The primer sequences used for PCR have been listed in Table 1. Amplification consisted of an initial 5 min incubation at 95°C and then 30 cycles of amplification using 30 s of denaturation at 95°C, 30 s at 56°C, and 60 s at 72°C. The final extension was set for 10 min at 72°C. All data were expressed as the relative differences between control and treated cells after normalization to β-actin expression.

**Table 1:** Primers used for semi-quantitative RT-PCR

Primer	Sequence	Length (bp)
Collagen III-F	5'-GGACCACCAGGGCCTCGAGGTAAC-3'	471

Collagen III-R	5'-TGTCCACCAGTGTTTCCGTG-3'	

Fibronectin-F	5'-TGGACCTTCTACCAGTGCGAC-3'	451

Fibronectin-R	5'-TGTCTTCCCATCATCGTAACAC-3'	

β-actin-F	5'-CCTCGCCTTTGCCGATCC-3'	626

β-actin-R	5'-GGATCTTCATGAGGTAGTCAGTC-3'	

### Protein extraction and western blotting

After the cells were grown and treated with or without TGF-β1, total cellular protein was extracted using a lysis buffer and quantified using protein quantification reagents from Bio-Rad. Next, 50 μg of the protein were suspended in 5x reducing sample buffer, boiled for 5 min, electrophoresed on 10% SDS-PAGE gels, and then transferred to polyvinylidene difluoride membrane by electroblotting. The membrane was blocked in 1% BSA/0.05% Tween/PBS solution overnight at 4°C, followed by incubation with the primary antibody (i.e., mouse monoclonal antibodies to either human fibronectin, collagen III, phosphorylated-Smad 2, 3, or total-Smad 2/3) for 24 h. A horseradish peroxidase-labelled goat anti-mouse IgG was used as the secondary antibody. The blots were then developed by incubation in a chemiluminescence substrate and exposed to X-ray films.

### Immunofluorescence staining

The expression of fibronectin in HMrSV5 cells was analyzed by Immunofluorescence microscopy. In brief, the cells were cultured on collagen-coated glass cover slips up to confluency and then fixed in 4% paraformaldehyde in 20 mM HEPES (pH 7.4) and 150 mM NaCl for 20 min. The glass cover slips were rinsed three times and permeabilized with 1.2% Triton X-100 for 5 min, rinsed three times again and then incubated with 1% BSA/0.05% Tween/PBS for 1 hour. Staining for expression of fibronectin was carried out with a primary rabbit antibody anti-fibronectin (1:200) and then with a secondary antibody conjugated with FITC. The DNA dye To-PRO-3 (blue) was used for counterstaining. The stained cells were mounted and viewed under immunofluorescence microscope.

### Tumor cell adhesion assay

The adhesion ability of gastric cancer cells to mesothelial cells was determined as described previously by Alkhamesi *et al*[18]. Briefly, HPMCs were grown in monolayer in 96-well plates overnight and treated with recombinant human TGF-β1 (5,10, 20 ng/mL) up to 72 h. Cancer cells were pretreated with or without the addition of 50 μg/ml RGD and stained with 15 μM of calcein AM for 30 min at 37°C and 5% CO_2_. Afterwards, these cells (5 × 10^4^/well) were added to the 96-well plates that contained peritoneal mesothelial cells and incubation occurred for 3 h at 37°C. The plates were then washed three times with 200 μl of growth medium to remove the non-adherent tumor cells. The remaining adherent tumor cells were observed under a fluorescence microscope and the total fluorescence in each well was recorded by a spectrofluorimeter using 485 nm and 535 nm wavelengths for excitation and emission, respectively. Another plate was seeded with labeled tumor cells for 3 h as positive control and its fluorescence intensity was considered as 100%. The adhesion percentage was calculated as follows:

% binding=100% ×(fluorescence intensity of the experimental group/positive control).

Prior to the experiments, the kinetics of binding of cancer cells were investigated. The peak adhesion of these cancer cells was observed after 3 h. For each group, the assay was performed in triplicate.

### Statistical analysis

All data were summarized as mean ± SE, where appropriate. The student's *t*-test was performed for the comparison of control and TGF-β1 treatment groups. Differences were considered statistically significant when the *p*-value was ≤ 0.05.

## Results

### Histological assessment of fibrosis reaction of the peritoneal tissues

**Figure 1:**
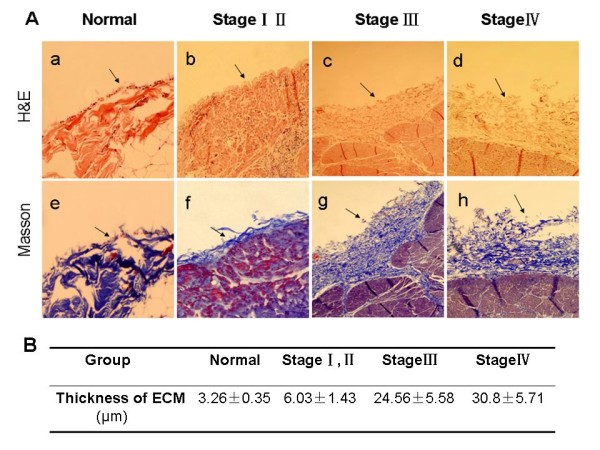
**Hematoxylin and eosin and Masson staining of peritoneum tissues**. Normal peritoneum and peritoneum from different stages of gastric cancer were obtained surgically and subjected to H&E and the Masson staining. **A**, All photos were obtained at 40 × magnification. a and e, The normal peritoneum consists of only a monolayer of mesothelium with little fibrosis (arrows). b and f, Peritoneum from the patients with early gastric cancer also showed small amounts of connective tissue under the mesothelial cells. In contrast, the peritoneum from patients with carcinomatosis at stage III (c, g) and IV (d, h) was substantially thickened and contained extensive fibrosis (arrows). **B**, Morphometric parameters of peritoneal tissues. Data are expressed as the mean ± standard error of the mean of at least 3 separate experiments.

We first stained and examined the histology of peritoneal tissues according to different gastric cancer stages for comparison with benign controls. As shown in Figure 1, the normal peritoneum consisted of only a monolayer of mesothelium cells with little connective tissue and the peritoneum from the patients with early gastric cancer also showed small amounts of connective tissue under the mesothelial cells. In contrast, the peritoneum from patients with carcinomatosis at stage III and IV was substantially thickened and contained extensive fibrosis. Most importantly, the peritoneal fibrosis was also found to occur in the peritonea from stage III gastric cancer in the absence of carcinomatosis.

### Detection of TGF-β1 levels in the peritoneal lavage fluid

**Figure 2:**
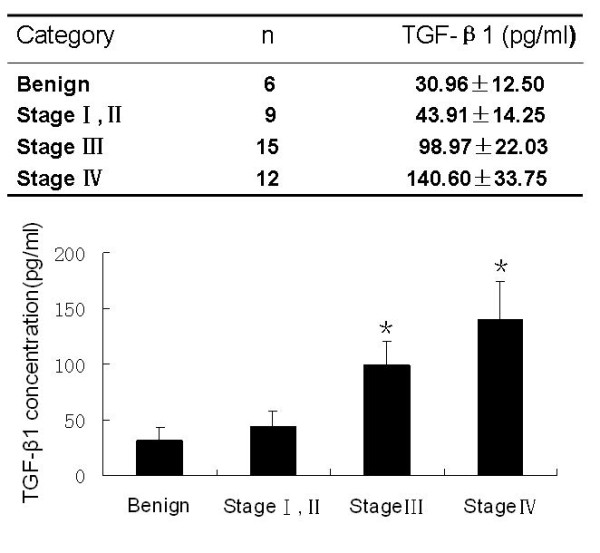
**ELISA analysis of TGF-β1 protein levels in peritoneal wash fluid**. Samples of the peritoneal wash fluid from patients with benign disease and gastric cancer were obtained and subjected to ELISA analysis. The data were summarized as mean ± standard error of the mean from at least 3 separate experiments. * *p *< 0.05 as compared with control.

We assayed TGF-β1 protein levels in the peritoneal wash fluid and found that TGF-β1 levels were significantly higher in those patients with gastric cancer than those in the control group. Levels were even higher in washes from patients with stage III or IV gastric cancer than those with stage I or II (Figure 2).

### Upregulation of collagen III and fibronectin expression by TGF-β1

**Figure 3:**
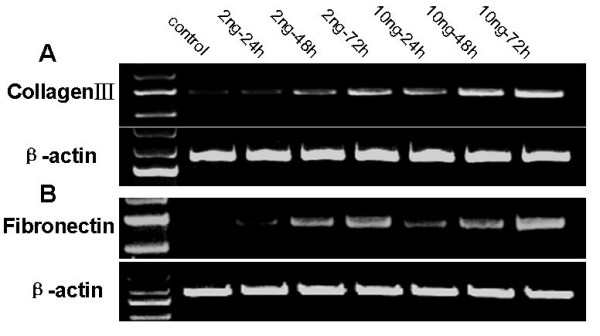
**Effects of TGF-β1 on expression of collagen III and fibronectin mRNA in HPMCs**. Serum-starved HPMCs were incubated with TGF-β1 (2 or 10 ng/ml) for up to 72 h and RNA was then isolated and subjected to semi-quantitative RT-PCR analysis of collagen III (A) and fibronectin (B). Expression of β-actin was used as a loading control.

**Figure 4:**
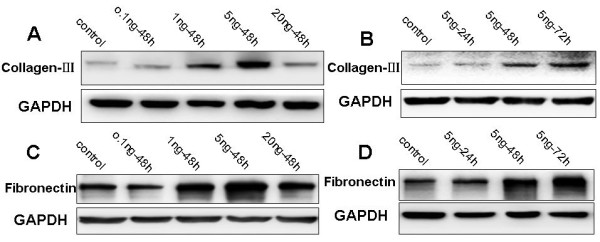
**Western blot analysis of collagen III and fibronectin protein levels in HPMCs with or without TGF-β1 treatment**. Serum-starved HPMCs were incubated with increasing concentrations of TGF-β1 for up to 72 h and total cellular protein was extracted and subjected to western blot analysis. **A**, Dose response of collagen III expression. **B**, Time course of collagen III expression. **C**, Dose response of fibronectin expression. **D**, Time course of fibronectin expression.

**Figure 5:**
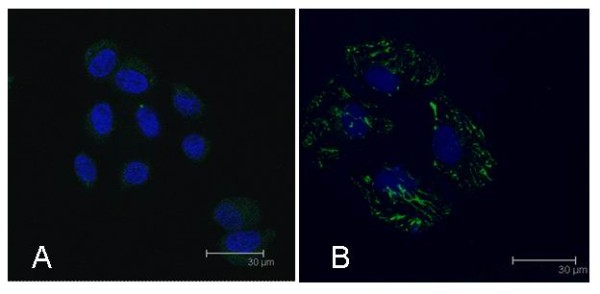
**Confocal immunofluorescence of fibronectin expression in mesothelial cells**. Serum-starved HPMCs were incubated with TGF-β1 for up to 72 h, and fixed for immunostaining with a polyclonal antibody against fibronectin. Fibronectin was visualized by FITC (green), and nuclei were visualized by To-PRO-3 (blue) under immunofluorescence confocal microscopy. **A**, Control cells. **B**, Mesothelial cells treated with TGF-β1 (5 ng/ml) for 72 h. All photos were obtained at 100× magnification.

We next determined whether TGF-β1 can affect extracellular matrix production (such as collagen III and fibronectin) in HPMCs. We cultivated and treated them with the recombinant human TGF-β1 and then performed semi-quantitative RT-PCR analysis. Our data showed that TGF-β1 upregulated expression of collagen III and fibronectin mRNA, as compared to the control group (*p *< 0.05) (Figure 3). Furthermore, Western blot analysis also confirmed this finding at the protein level (Figure 4). In addition, we use immunofluorescence microscopy to confirm this protein data (Figure 5); TGF-β1-treated HPMCs exhibited a fibrillar pattern of fibronectin-specific proteins after 48 h treatment that was markedly absent in the controls.

### TGF-β1 induction of Smad 2 and 3 phosphorylation in HPMCs

**Figure 6:**
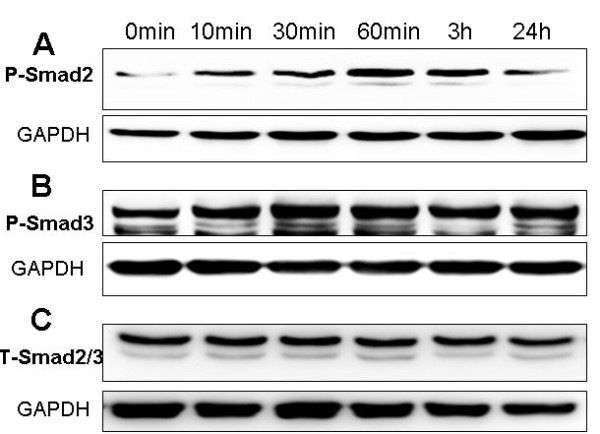
**Effects of TGF-β1 on Smad 2 and 3 phosphorylation in the mesothelial cells**. The HPMCs were grown in serum-free medium with or without 5 ng/mL TGF-β1 treatment for up to 24 h. Total cellular protein was then extracted and subjected to Western blot analysis. **A**, Expression of phosphorylated Smad 2 protein. **B**, Expression of phosphorylated Smad 3 protein. **C**, Total Smad 2/3 protein.

To determine how TGF-β1 regulates collagen III and fibronectin expression, we treated HPMCs with 5 ng/ml of TGF-β1; subsequent western blot analysis showed that TGF-β1 induced phosphorylation of Smad 2 and 3 starting at 10 min post-treatment and reached a maximum between 30-60 min, but TGF-β1 did not affect the total Smad 2 and 3 expression levels (Figure 6).

### Induction of gastric cancer cell adhesion to the mesothelial cells through peritoneal fibrosis

**Figure 7:**
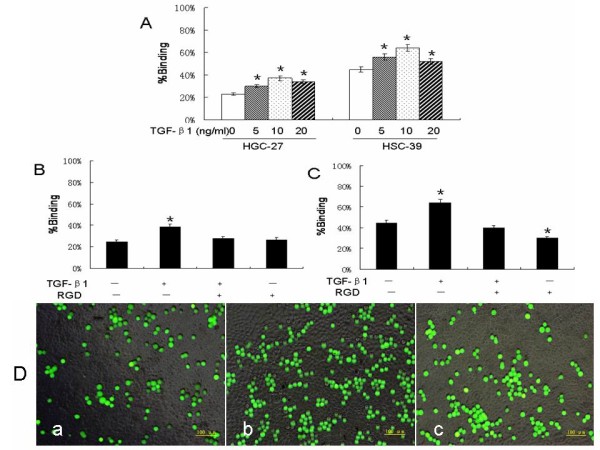
**Effects of TGF-β1 and RGD on adhesion of gastric cancer cells to mesothelial cells**. **A**, Gastric cancer HGC-27 and HSC-39 cells were grown and treated with or without TGF-β1 (5, 10, 20 ng/ml) for 48 h and then subjected to cell adhesion assay. **B**, HPMCs were incubated with TGF-β1 and HGC-27 cancer cells were pretreated with or without RGD, and then cancer cells were added onto the mesothelial cell culture and subjected to cell adhesion assay. **C**, HPMCs were incubated with TGF-β1 and HSC-39 cancer cells were pretreated with or without RGD, and then cancer cells were added onto the mesothelial cell culture and subjected to cell adhesion assay. **D**, Fluorescence microscopy (x 40) of gastric cancer HGC-27 cells adhered to the confluent mesothelial cells. a, mesothelial cells without TGF-β1 treatment; b, mesothelial cells treated with 5 ng/ml TGF-β1 for 48 h; c, gastric cancer HGC-27 cells were pretreated with RGD, and then added onto the mesothelial cells that were pretreated with TGF-β1 (5 ng/ml) for 48 h. * *p *< 0.05 as compared with control.

We then assessed the role of peritoneal fibrosis and RGD (Arg-Gly-Asp sequences) in regulating the adhesion ability of gastric cancer cells to mesothelial cells. Through fluorescently examining the level of tumor cells adhering to mesothelial cells in response to TGF-β1 treatment, we found that peritoneal fibrosis appeared to be able to promote gastric cancer cell adherence to mesothelial cells in a TGF-β1 dose-dependent manner, as compared to the control (*p *< 0.05). RGD decreased the number of cancer cells to adhere to the mesothelial cells under TGF-β1 stimulation (Figure 7). The data on cancer cells obtained from ascites or no-ascites also showed similar results.

## Discussion

In the current study, we first assessed the histology of peritoneal tissues and detected the TGF-β1 levels in peritoneal wash fluids obtained from patients with gastric cancer and benign disease. After that, we determined the role of TGF-β1 in promotion of collagen III and fibronectin expression and then performed tumor cell adhesion assay to identify the effects of TGF-β1 on the mesothelial cells, as well as on Smad 2 and 3 expression. We found that the peritoneum was significantly thickened in gastric cancer patients and consisted of extensive fibrosis; in addition, TGF-β1 levels were also dramatically increased in peritoneal wash fluid from stage III or IV gastric cancer compared to that from stage I and II gastric cancer and benign disease. TGF-β1-treated mesothelial cells exhibited increased collagen III and fibronectin expression and promoted gastric cancer cells adherence to mesothelial cells. It has been hypothesized that the effects of TGF-β1 may be mediated by induction of Smad 2 and 3 phosphorylation in the mesothelial cells. The data from the current study indicate that induction of peritoneal fibrosis by TGF-β1 may provide a suitable environment for the dissemination of gastric cancer. The interaction of gastric cancer with peritoneal mesothelial cells could provide the theoretical 'seed' and 'soil' to promote gastric cancer metastasis to the peritoneum.

It is generally believed that gastric cancer occupies a unique position to metastasize to the peritoneum, due to its ability to readily physically invade into the peritoneal cavity. However, a more complicated process may be involved. For example, the peritoneal microenvironment may also favor implantation of gastric cancer cells on the peritoneal lining [7]. Attachment of malignant cells to the peritoneal mesothelium is thought to be a critical step in peritoneal dissemination of the disease [19]. Previous studies have suggested that this process was mediated by interaction between extracellular matrix and the corresponding adhesion molecules from gastric cancer cells [20]. Moreover, the extracellular matrix may serve to anchor the cancer cells [9]. Indeed, our current study has demonstrated such an interaction and showed that TGF-β1 promoted the peritoneal fibrosis that in turn provided a suitable 'soil' for metastasis. We found that the peritoneum from patients with stage III and IV gastric cancer and peritoneal carcinomatosis was thickened and consisted of extensive fibrosis and mass stroma cell infiltration. Most importantly, fibrosis also occurred in the peritonea from the stage III gastric cancer tissues even in the absence of carcinomatosis, indicating that this peritoneal fibrosis did not depend on tumor presence but instead may have been promoted by inflammatory factors, such as TGF-β1, secreted by gastric cancer cells [21].

The cause of peritoneal fibrosis in gastric cancer patients has been investigated previously, and TGF-β1 was identified as one of the most potent fibrotic stimuli for mesothelial fibrosis [22,23]. For example, our previous study showed that TGF-β1 expression in gastric cancer tissues was closely associated with the depth of gastric cancer cell infiltration and peritoneal metastasis of gastric cancer. But, it was unclear how TGF-β1 induced gastric cancer cell invasion and metastasis to the peritonea. Our current study indicated that the induced TGF-β1 level observed in the peritoneal wash fluid could play a key role in promoting peritoneal fibrosis and create a suitable environment for gastric cancer metastasis. This idea was further supported by gastric cancer cell adhesion assay that showed TGF-β1-treated peritonea were more favorable for gastric cancer cell adhesion. In addition, we also observed that the levels of TGF-β1 were closely related to the degree of peritoneal fibrosis in gastric cancer patients (Stage III and IV gastric cancers had high levels of TGF-β1 in the peritoneal wash fluid, but also had more extensive peritoneal fibrosis). The data suggested that TGF-β1 secreted by gastric cancer cells was able to promote peritoneal fibrosis and in turn provide suitable 'soil' for metastasis.

In order to confirm the effect of TGF-β1 on peritoneal fibrosis, we showed that TGF-β1 affected the function of mesothelial cells by stimulating extracellular matrix (including fibronectin and collagen III) production, which consists of molecules important in cell adhesion and tissue repair [24,25]. TGF-β1 induced fibronectin and collagen III expression in both dose- and time-dependent manners. Meanwhile, immunolocalization showed that expression of fibronectin protein was induced by TGF-β1 in HPMCs. These data further supported the central role theory for TGF-β1 in peritoneal fibrosis and may provide a useful model by which to study peritoneal metastasis of gastric cancer.

It is well known that TGF/Smad pathway can regulate multiple cellular functions including inhibition and stimulation of cell growth, cell death or apoptosis and cellular differentiation [26-28]. In the current study, we demonstrated that TGF-β1 was able to induce Smad 2 and 3 phosphorylation in HPMCs. These data indicated that rapid and sustained phosphorylation of Smad 2 and Smad 3 may participate in TGF-β1-induced peritoneal fibrosis.

Many studies have investigated the impact of the cancer-stroma interaction in different human cancers and shown the importance of tumor cell interaction with extracellular matrix to establish a favorable microenvironment for tumor cell growth, invasion, and metastasis [18,29,30]. Our data from the current study confirmed such an interaction, in that TGF-β1 secreted by gastric cancer cells was able to increase production of fibronectin and collagen III in HPMCs and in turn induce peritoneal fibrosis. TGF-β1-treated mesothelial cells affected gastric cancer cell adhesion. We also determined whether these effects are ECM-dependent by using RGD to achieve selective and specific knockdown of minimal sites of ECM cell binding domain. We found that RGD treatment significantly decreased the adhesive ability of cancer cells to mesothelial cells. These data suggest that peritoneal fibrosis may stimulate the adherence capability of gastric cancer cells to the peritoneum, which is consistent with previous reports showing that TGF-β1 enhanced tumor-mesothelial cell adhesion [31,32].

We have also noticed that the concentration of TGF-β1 in the peritoneal wash fluid was lower than that to use *in vitro* to treat mesothelial cells. It may the natural differences between *in vivo* and *in vitro* experiments and the latter is acute and artificial. In addition, some other factors secreted by gastric cancer cells may also contribute to the effect.

In conclusions, our current study characterized the interaction of gastric cancer with peritoneal fibrosis and determined that TGF-β1 plays a key role in induction of peritoneal fibrosis, which in turn affected gastric cancer adhesion and metastasis. Furthermore, the pretreatment of cancer cells with RGD significantly inhibited the adhesion of carcinoma cells. Taken together, our current data demonstrated that the presence of peritoneal fibrosis appears to provide a favorable environment for dissemination of gastric cancer.

## Competing interests

The authors declare that they have no competing interests.

## Authors' contributions

ZDL, DN, FNL and ZMD participated in most of the experiments. ZS and XYM participated in the design of the study and performed the statistical analysis. ZDL and ZL collected tissue specimens and drafted the manuscript. HMX and ZNW conceived of the study, and participated in its design and coordination. All authors read and approved the final manuscript.
